# Metastasis of Gonorrhœa

**Published:** 1894-03

**Authors:** I. Dever

**Affiliations:** Clinton, N. Y.


### METASTASIS OF GONORRHŒA.

I. Dever, M. D., Clinton, N. Y.

The case of gonorrhœa with treatment, and complications resulting from the treatment found on page 62 of February number of Homœopathic Physician, extracted from *The Medical News,* is but a fair sample of allopathic bungling to be found in any allopathic journal of the day. And right sorry am I to say that such treatment of gonorrhœa and other acute difficulties are too often found in homœopathic journals—reported by homœopathic pretenders, who claim wonderful progress in the treatment of all forms of disease, and point to such treatment as an example to all who would meet with the fullest measure of success.

Now, with your permission, I will report a different course of treatment, with somewhat different results from those obtained by the treatment prescribed in *The Medical News.* About the first of last December, I was called to see a young man who had been in poor health for six weeks, as he informed me. I put him through a course of questioning, but on all points which would throw any light on my suspicions he would shut up tight as a clam. Not one word could I get out of him leading to the cause of his peculiar symptoms. He was pale, thin, and nervous; had great pain in his head and neck, and would have paroxysms of palpitation, when the perspiration would stand out all over him in great drops. He could not rest in any position; he was thirsty, and kept in continual motion. When still, he would hold his head with both hands. His tongue was dry and cracked—somewhat pointed, and he could not eat, sleep, or be quiet in any position—yet he was very weak and trembled from head to foot. I prescribed Rhus$^{2c}$, one dose, with some benefit. I followed it up in a week with Rhus$^{cm}$. He improved, but slowly. One day I happened to meet his younger brother, who said to me: "Say! did my brother tell you that he had had the clap all last summer, and that he had just got cured when he was taken with the pain in his head?"

I hitched up and went to see that young man. I told him that I had waited long enough on him to be entitled to an explanation of the cause of his severe difficulty, which, though it showed symptoms which he was pleased to call rheumatic, yet I knew it was the result of the gonorrhœa which he had contracted the previous summer, and for which he had taken injections of Zinc and other vile mixtures. He then gave me the following history: "I contracted the gonorrhœa last summer, but Dr. —— cured me of it all, with the exception of a thin watery discharge, which he could not cure until he gave me a white powder, with directions to dissolve it in four ounces of water and inject it into the penis three times every day."

When I heard this I had no further doubt of the nature of my case. It was a pure and unadulterated case of metastasis.

I at once prescribed one dose of Medorrhinum$^{cm}$, and notwithstanding his weak condition, he lost his pain and gained right along from the time that he received the medicine, and in two weeks was at work on the farm.

I would here say that I found a picture of my case in the provings of Medorrhinum, Vol. 7, Hering's *Guiding Symptoms.*

